# Errors Affect Hypothetical Intertemporal Food Choice in Women

**DOI:** 10.1371/journal.pone.0108422

**Published:** 2014-09-22

**Authors:** Manuela Sellitto, Giuseppe di Pellegrino

**Affiliations:** 1 Dipartimento di Psicologia, Università di Bologna, Bologna, Italy; 2 Centro studi e ricerche in Neuroscienze Cognitive, Polo Scientifico-Didattico di Cesena, Cesena, Italy; Università di Trento, Italy

## Abstract

Growing evidence suggests that the ability to control behavior is enhanced in contexts in which errors are more frequent. Here we investigated whether pairing desirable food with errors could decrease impulsive choice during hypothetical temporal decisions about food. To this end, healthy women performed a Stop-signal task in which one food cue predicted high-error rate, and another food cue predicted low-error rate. Afterwards, we measured participants’ intertemporal preferences during decisions between smaller-immediate and larger-delayed amounts of food. We expected reduced sensitivity to smaller-immediate amounts of food associated with high-error rate. Moreover, taking into account that deprivational states affect sensitivity for food, we controlled for participants’ hunger. Results showed that pairing food with high-error likelihood decreased temporal discounting. This effect was modulated by hunger, indicating that, the lower the hunger level, the more participants showed reduced impulsive preference for the food previously associated with a high number of errors as compared with the other food. These findings reveal that errors, which are motivationally salient events that recruit cognitive control and drive avoidance learning against error-prone behavior, are effective in reducing impulsive choice for edible outcomes.

## Introduction

There is increasing incidence of eating disorders, frequently resulting in obesity or other chronic weight problems (e.g., [1]–[4]). These abnormal behaviors reflect exceeding appetitive motivation to obtain and consume palatable edible reinforcers immediately (frequently sweet high-fat foods, e.g., [5], [6]), over long-term benefits of following a healthy diet, which requires capacity for eating control [7], [8]. The ability to forgo sooner gratification in favor of delayed reward is crisply captured by intertemporal choice paradigms (e.g., [9]), in which decisions involve smaller-immediate rewards vs. larger-later rewards (e.g., [10], [11]). Usually, the subjective value (i.e., the utility depending on specific characteristics of a subject) of potential reward is weakened (discounted), as a function of the time until its delivery (the so-called delay or temporal discounting phenomenon; e.g., [12]–[17]). Suboptimal intertemporal choice (i.e., increased sensitivity to immediate temptations and increased temporal discounting) is a core feature of impulsivity (e.g., [18]) and it has been related to several pathological conditions, including drug addiction and obesity (e.g., [18]–[27]). Individuals suffering from eating disorders have enhanced attentional bias toward food cues [28], [29], as well as increased reinforcing value placed on food [30]. However, the simple visual exposure to tempting food is a powerful trigger for immediate consumption even in healthy population [31], [32].

In order to resist successfully to immediate gratification in the service of a long-term good, one must be able to control the self and refrain one’s natural impulse to consume now. Self-control is clearly related to cognitive control, and involves the capacity individuals have to override or alter their predominant response tendencies, thereby allowing other more appropriate responses [33]. But how a person knows when to recruit self-control strategies? Both theoretical perspectives and empirical findings suggest that the ability to control behavior is enhanced in contexts in which errors are more frequent [34]. Errors are typically highly arousing, aversive events that elicit compensatory responses [35]. Importantly, errors represent lapses in performance (and utility) and signal the need to augment on-line the top-down control for the current and future behaviour [36]. Indeed, they drive avoidance learning against error-prone, maladaptive responses and thereby they contribute to the optimization of decision-making performance [37]–[40]. As aversive events, errors produce a negative neural wave (error-related negativity, ERN; [41], [42]), generated in the anterior cingulate cortex (ACC) (e.g., [43]), both when subjects become aware of having committed a mistake and when they are not explicitly aware of making the error [43]–[44]. It has been suggested that the ERN may signify affective processing in response to errors [45], and individuals with larger ERNs showed enhanced avoidance learning for events associated with negative outcomes ([39]–[40], [46]; see also [47]).

Here we investigated whether pairing desirable foods with errors could signal a performance-related cost (i.e., a loss in reward) and drive increased compensatory self-control strategies, thereby reducing impatient food choices [39], [47]. To this aim, participants performed a Stop-signal task (adapted from [48]) in which two different foods served as cues predicting different error likelihoods in performance, high and low, respectively. Following the Stop-signal task, we measured participants’ intertemporal preference for the two food items using separate Temporal Discounting tasks (adapted from [49]). More specifically, participants decided between smaller amounts of one food available immediately and larger amounts of the same food delivered after a variable delay. We expected reduced impulsive preferences for the food associated with the high-error rate as compared to the food associated with the low-error rate. Furthermore, we controlled for participants’ hunger since it is known to influence the immediate evaluation of food items (e.g., [50]–[53]) by increasing food reinforcing value and, consequently, the discounting of future amounts of rewards [7], [54]–[56].

Finally, since recent findings highlighted that women and men have different behavioral (e.g., [57]–[58]) and neural (e.g., [59]–[61]) responses to food cues, hunger, and satiation, we tested female participants only, to control for sources of variability.

In line with our prediction, results showed that pairing food with errors reduced impulsive choices for the food associated with the high-error rate compared with the food associated with the low-error rate, by controlling for hunger level. This result indicates that the aversive nature of errors, by signalling increased need of adaptive strategies, can be transferred on food when it has to be chosen, and that this effect is modulated by the food-related motivational state of participants. That is, participants with low level of hunger showed reduced impulsive preference for the food previously associated with a high number of errors as compared with the other food, whereas participants with higher level of hunger failed to reveal difference in the TD rates for the two foods.

## Materials and Methods

### Ethics statement

The study involved healthy young adult females. All subjects gave written informed consent according to the ethical guidelines of the Declaration of Helsinki [62]. The institutional Ethical Committee of the Department of Psychology of the University of Bologna specifically approved this work.

### Participants

Forty young adult females took part in the study (see Table 1 for demographic information). All participants were not taking psychoactive drugs, they were not on a diet, and they were free of current or past psychiatric or neurological illness as determined by history. Subjects remained naïve as to the purpose of the study until debriefing, at the end of the experimental session.

**Table 1:** Participants’ demographic data.

N	Age	Education	BMI	Hunger	Fasting	DEBQ_EEB_
40	25 (0.6)	16 (0.3)	21 (0.5)	1.7 (0.5)	4 (0.5)	3 (0.1)

BMI = Body Mass Index; DEBQ_EEB_ = External Eating Behavior subscale of the Dutch Eating Behavior Questionnaire. Age and education are expressed in years. BMI is expressed in kg/m^2^. Fasting is expressed in hours. Numbers in parenthesis are standard errors.

### Procedure

After collecting participants’ demographics (Table 1), including height and weight in order to calculate their body mass index (BMI; [63]–[64]), subjects first rated their hunger level and their willingness to eat at the time of the experiment six foods depicted in picture. Following the rating session, participants performed a Stop-signal task and, afterwards, two Temporal Discounting tasks. At the end, participants filled out the External Eating Behavior subscale of the Dutch Eating Behavior Questionnaire (DEBQ_EEB;_ [65]).

### Ratings

#### Hunger

Participants rated on 11-points scale their hunger level at the time of the experiment (*baseline hunger*, from −5, “not hungry at all”, to 5, “extremely hungry”).

#### Food

Following the hunger rating, participants rated on 11-points scale the willingness to eat at the moment each of six sweet food items, one at a time (*wanting*, from −5, “not at all”, to 5, “extremely”). Foods were presented in high-resolution color pictures (150 dpi), matched for dimension, luminance, and contrast, on a 15-inch computer screen located approximately 40 cm away from the participants. There were no discrepancies between the size of objects depicted on the screen and their real size in the world. All pictures were presented in a random order across participants.

### Stimuli selection

Following the rating session, the experimenter chose, individually for each participant, the two foods that obtained the higher score. These two foods were used as stimuli in the following Stop-signal task and in the two Temporal Discounting tasks. Since these two foods obtained often a different evaluation, they were assigned in a counterbalanced order to the two different error conditions of the Stop-signal task (see below for details). We compared stimuli ratings using nonparametric statistics since they were not normally distributed.

### Stop-signal task

Based on [48]’s paradigm, a Stop-signal task was adjusted using as cues the two foods previously selected after the rating session. Two types of conditions were included. One condition had a low-error likelihood (Low-Error condition), and the other condition had a high-error likelihood (High-Error condition). One food (Low-Error Food, LEF) was used as cue for the Low-Error condition; the other food (High-Error Food, HEF) was used as cue for the High-Error condition. Hence, for a given trial, food type predicted low- versus high-error likelihood in performance, respectively.

**Figure 1:**
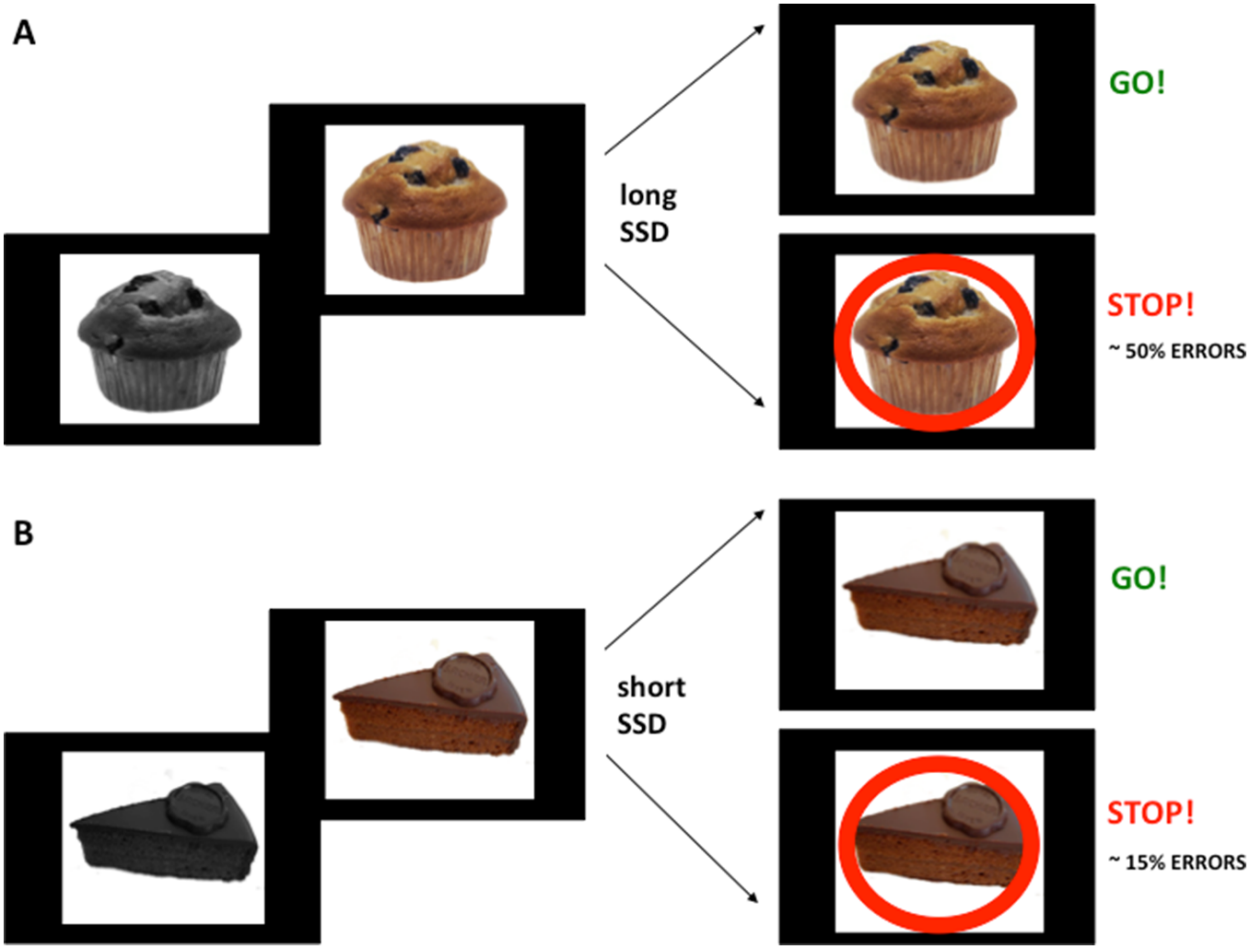
Stop-signal task experimental paradigm. In this trial example, the muffin (A) is assigned to the High-Error condition, whereas the chocolate cake (B) is used as cue for the Low-Error condition. Each trial began with a black and white picture displayed for 1000 ms. Then, the black and white picture became coloured: This represented the Go signal. On 33% of the trials, a Stop signal (a red circle) was postponed to the Go signal after a variable SSD relative to the Go signal onset. The High-Error condition (A) had longer SSDs. The Low-Error condition (B) had shorter SSDs. Both Go and Stop signals remained visible until a response deadline of 1000 ms after Go signal onset in both error conditions.

Figure 1 illustrates the experimental paradigm. Each trial began with a black and white picture of one of the two foods appearing for 1000 ms. Later on, the black and white picture became coloured, thus prompting the Go signal. The Go signal required participants to press the down arrow button on the keyboard as quickly as possible. However, on 33% of the trials, a Stop signal was postponed to the Go signal after a variable Stop-Signal Delay (SSD) relative to the Go signal onset. This Stop signal, a red circle appearing around the coloured picture, indicated that the response to the Go signal was no longer required. Both Go and Stop signals remained visible until a response deadline of 1000 ms after Go signal onset, which indicated a time limit of 1000 ms to produce or not a response. After each trial, a blank screen appeared for a variable intertrial interval (500, 1000, 1500, 2000 ms). Error rates (low vs. high) were explicitly set and controlled by dynamically adjusting the SSDs for each error likelihood condition independently with the use of a staircase algorithm. The Low-Error condition had shorter SSDs, whereas the High-Error condition had longer SSDs [48]. The SSD started at 200 ms (after Go signal onset) for both Low- and High-Error conditions. During the Low-Error condition, if the participant succeeded in withholding the response after the Stop signal, the SSD increased by only 5 ms; conversely, if the subject failed, the SSD decreased by 50 ms on the next trial. During the High-Error condition, if the participant succeeded in withholding the response after the Stop signal, the SSD increased by 50 ms; conversely, if the subject failed, the SSD was decreased by 50 ms on the next trial [66].

After a correct response to the Go signal or a non-response to the Stop signal, the message “Correct!” (in Italian) appeared on the screen. If participant did not respond in time to the Go signal, or if she responded to the Stop signal, a message “Error!” appeared on the screen. If participant responded to the black and white picture, a message “Too early!” appeared on the screen. Trials pertaining to the Low-Error condition and the High-Error condition were released in a random order. All cues and stimuli were presented centrally on a black background.

Participants performed 240 trials in total: 160 Go trials (80 for the Low-Error condition and 80 for the High-Error condition) and 80 Stop trials (40 for the Low-Error condition and 40 for the High-Error condition). The task lasted approximately 16 minutes.

#### Stop-signal task data analysis

We performed data analysis on the accuracy (number of errors) and the RTs of correct responses vs. errors (no-response trials ignored), for the Low- vs. High-Error condition, and for Go vs. Stop trials [48].

### Temporal discounting task

After the Stop-signal task completion, participants underwent to two Temporal Discounting (TD) tasks, administered separately in a counterbalanced order across participants. In each of the two computerized TD tasks, participants performed a series of intertemporal choices. In each trial, they chose between a smaller amount of bites (units) of a hypothetical food [49], [67]–[68] that could be received immediately and a larger amount of bites of the same food that could be received after some specific delay (e.g., [69]–[70]). One task assessed subjective preferences in time for LEF, and one task assessed subjective preferences in time for HEF. Both LEF and HEF were preselected during the rating session and were the same as in the Stop-signal task.

In each task, participants made five choices at each of six delays: 2 days, 2 weeks, 1 month, 3 months, 6 months, and 1 year. The order of blocks of choices pertaining to different delays was randomly determined across participants. Within each block of five choices, the delayed amount was always 40 units [49]. The amount of the immediate reward, on the other hand, was adjusted based on the participant’s choices, using a staircase procedure that converged on the amount of the immediate reward that was equal, in subjective value, to the delayed reward [49]. The first choice was between a delayed amount of 40 units (e.g., 40 bites or tastes of muffin) and an immediate amount of 20 units (e.g., 20 bites of muffin). If the participant chose the immediate reward, then the amount of the immediate reward was decreased on the next trial; if the subject chose the delayed reward, then the amount of the immediate reward was increased on the next trial. The size of the adjustment in the immediate reward decreased with successive choices: the first adjustment was half of the difference between the immediate and the delayed reward, whereas for subsequent choices it was half of the previous adjustment [70]. This procedure was repeated until the subject had made five choices at one specific delay, after which the subject began a new series of choices at another delay. For each trial in a block, the immediate amount represents the best guess as to the subjective value of the delayed reward. Therefore, the immediate amount that would have been presented on the sixth trial of a delay block was taken as the estimate of the subjective value of the delayed reward at that delay (see also [49]).

**Figure 2:**
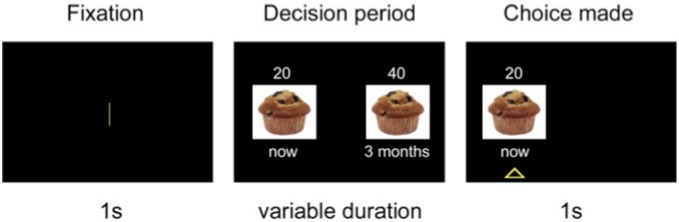
Temporal discounting task experimental paradigm. In each trial, after a 1000 ms fixation period, subjects chose between a small amount of reward delivered immediately and a larger amount of reward delivered after a delay. The preferred option remained highlighted for 1000 ms. Food offered was hypothetical.

Participants were told that, on each trial, two amounts of hypothetical reward would appear on the screen. One could be received right now, and one could be received after a delay. They were explicitly asked to imagine receiving the two amounts of reward, and they were required to indicate the option they preferred by pressing one of two buttons [49], [71]. They were informed that there were no correct or incorrect choices. Figure 2 illustrates the experimental paradigm. Each trial began with a 1 s fixation screen, followed by a screen depicting the two available options. The two options appeared on the left and right side of the screen, and clearly indicated the food type, the amount of reward, and the delay of delivery of the reward. After the participants made their choices, the non-preferred option disappeared, whereas the preferred option remained on the screen for 1 s, with a triangle underneath it. The intertrial interval was 1.5 s.

#### TD data analysis

For each task, the rate at which the subjective value of a reward decays with delay (TD rate) was assessed through the TD parameter (*k*) [72]–[74]. The hyperbolic function SV = 1/(1+*k*D), where SV = subjective value (expressed as a fraction of the delayed amount), and D = delay (in days), was fit to the data to determine the *k* constant of the best fitting TD function, using a nonlinear, least-squares algorithm (as implemented in Statistica; Statsoft). The larger the value of *k*, the steeper the discounting function, the more participants were inclined to choose small-immediate amounts over larger-delayed amounts.

Moreover, for comparison purposes, we also assessed the fits to the data of an exponential discounting model. For each TD task, the exponential function SV = e^−*k*D^ was fit to the data to determine the *k* constant of the best fitting TD function, using the same procedure as for the hyperbolic function.

### DEBQ_EEB_

This subscale ([65]; Italian version, [75]) measures the sensitivity to external edible cues, such as during food exposure, as component of eating behaviour [65]. Participants reported for each item the frequency of engagement to the described behaviors on 5-points scale. Higher scores correspond to higher sensitivity to external cues, such as sight and smell of food [65] (see Table 1).

## Results

### Stimuli selection

The two food cues selected after the rating session had equivalent *wanting* ratings across participants (Wilcoxon signed-rank test, T = 186, p = 0.94).

### Stop-signal task

#### Accuracy

An ANOVA on percentage of errors with condition (Low-Error, High-Error) and trial type (Go, Stop) as within subject factors yielded a significant effect of condition (F(1, 39) = 1537.53, p = 0.000001), a significant effect of trial type (F(1, 39) = 844.54, p = 0.000001), and a significant interaction condition×trial type (F(1, 39) = 1122.91, p = 0.000001). Post hoc comparisons, performed with the Newman-Keuls test, showed that the percentage of errors was significantly higher during the High-Error condition than the Low-Error condition (28% vs. 11%; p = 0.0001), and that the number of errors committed during No-go trials was significantly higher than the number of errors committed during Go trials (32% vs. 7%; p = 0.0001). Moreover, while the percentage of errors committed during Go trials was the same for both High and Low conditions (7.07% vs. 7.09%; p = 0.98), the percentage of errors during No-go trials was significantly higher for the High-Error condition than the Low-Error condition (50% vs. 15%; p = 0.0001).

#### RTs

An ANOVA on RTs for correct trials with condition (Low-Error, High-Error) and trial type (Go, Stop) as within subject factors evidenced a significant effect of condition (F(1, 39) = 40, p = 0.000001), a significant effect of trial type (F(1, 39) = 420, p = 0.000001), and a significant interaction condition×trial type (F(1, 39) = 45.5, p = 0.000001). Post hoc comparisons, performed with the Newman-Keuls test, showed that RTs were significantly higher (i.e., responses were slower) during the High-Error condition than the Low-Error condition (430 ms vs. 405 ms; p = 0.0001), and that RTs during Go trials were significantly higher than RTs during No-go trials (454 ms vs. 381 ms; p = 0.0001). Moreover, while RTs for correct responses during Go trials were the same for both High and Low conditions (455 ms vs. 453 ms; p = 0.99), RTs for correct responses during No-go trials were significantly higher for the High-Error condition than the Low-Error condition (404 ms vs. 357 ms; p = 0.0001).

The above results, in line with previous findings (e.g., [48]), support the validity of the Stop-signal paradigm in producing different patterns of errors, and are indicative of the higher conflict experienced during the High-Error than in the Low-Error condition. Indeed, participants made clearly a significantly higher number of errors during the High-Error condition when performing Stop trials. These effects were apparently implicit: at debriefing, subjects were mostly unaware of the error manipulation even when directly prompted. This might be explained by the presentation of trials and error-conditions, which were all interspersed randomly, and by the highly demanding nature of the task, thereby requiring participants to stay focused in order to perform the task as much accurately as possible.

### TD task

**Figure 3:**
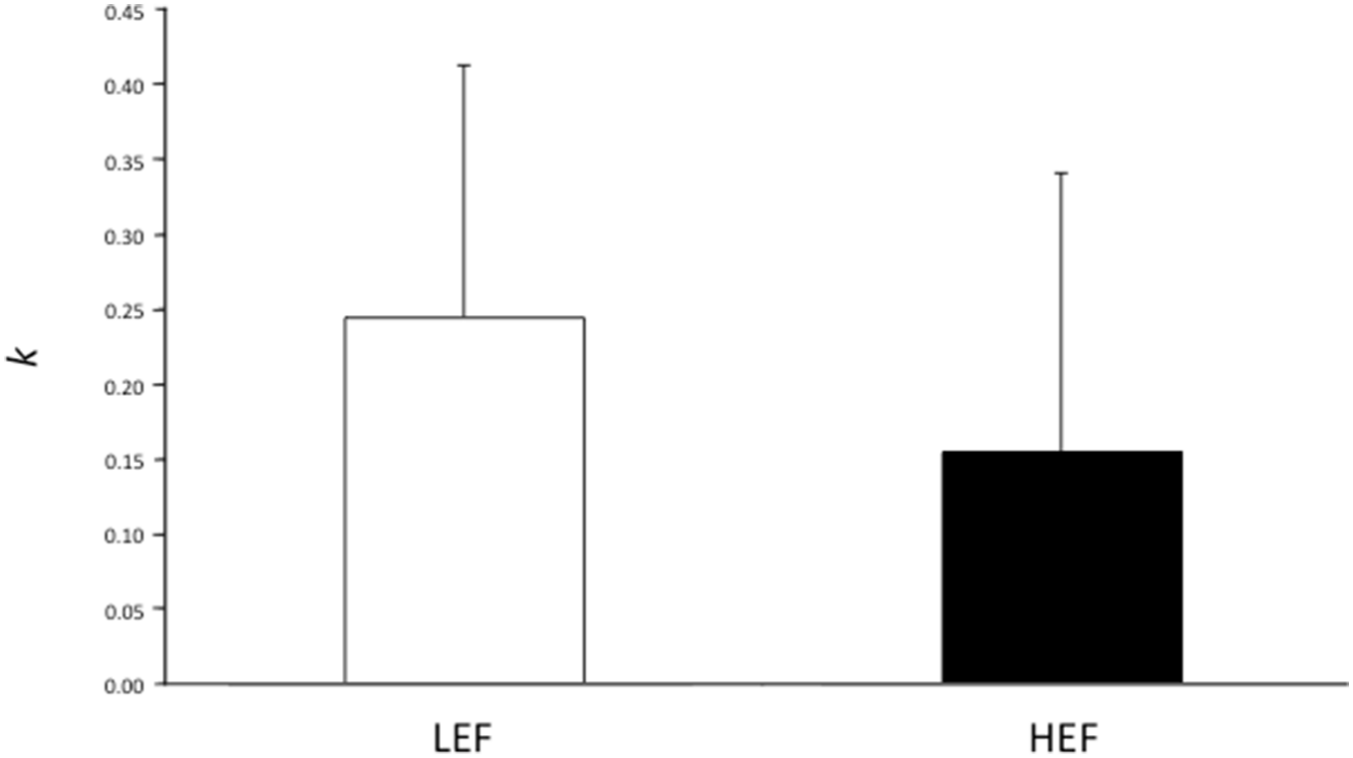
TD rates by food type. Bars reflect the geometric mean of TD rate *k* for LEF and HEF respectively. The smaller the bar, the smaller the TD rate, the smaller the number of impulsive choices. The error bars indicate the SEM.

Figure 3 shows TD rate by food type (LEF and HEF). Bars reflect the geometric mean of the TD rate for each food cue – which corresponds to mean of the log-transformed values – and thus provides a better measure of central tendency for positively skewed metrics, such as TD rates, than does the arithmetic mean [49]. We chose bars rather than curves to represent TD rates since *k* values were quite small (e.g., [71], [76]–[77]), in order to visually reveal better the difference between the TD rates for the two foods. The smaller the bar, the smaller the TD rate (less steep discounting), the smaller the number of impulsive choices.

First, subjective preferences were well characterized by hyperbolic functions, with no significant differences in R^2^ between LEF and HEF (LEF: 0.68 vs. HEF: 0.65; F(1, 39) = 0.26, p* = *0.61). The absence of differences in R^2^ in describing discounting behavior applied to foods associated differently to error likelihoods indicates that error rates did not alter TD in its shape. Moreover, although both the hyperbolic and the exponential functions fit the data well, the hyperbolic function fit better than the exponential across foods. Indeed, when we entered hyperbolic and exponential R^2^ scores as the dependent variables in an ANOVA for each food separately, the hyperbolic model fit better than the exponential model for both the LEF (0.68 vs. 0.58; F(1, 39) = 31.55, p = 0.000002), and the HEF (0.65 vs. 0.59; F(1, 39) = 18.89, p = 0.0001). Given the superiority of the hyperbolic over the exponential model in describing discounting behavior, hyperbolic *k* values were adopted as measures of TD [49].

Second, the hyperbolic k constants were normally distributed after log-transformation (Kolmogorov–Smirnov d<0.14, p>0.2 in all cases), and therefore, comparisons were performed using parametric statistical tests.

Thus, hyperbolic *k* values were entered in an ANOVA, with food type (LEF, HEF) as within subject factor, and *baseline* hunger as covariate to control for hunger state. We found a significant effect of food type (F(1, 38) = 7.65, p = 0.01), a significant effect of *baseline* hunger (F(1, 38) = 6.53, p = 0.01), and a significant food type×*baseline* hunger interaction (F(1, 38) = 7.73, p = 0.01). Post-hoc comparisons, performed with the Newman-Keuls test, indicated that HEF was discounted significantly less than LEF (−0.81 vs. −0.61), as determined by hunger level. Please, note that the results of this analysis on TD rates for LEF and HEF remained unaffected by including as covariates BMI and DEBQ_EEB_ scores as well. No significant interaction between these two covariates and the main effect emerged (F(1, 39)<1.5, p>0.23 in all cases).

**Figure 4:**
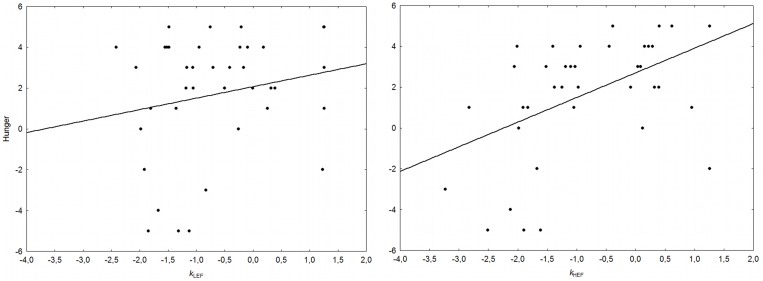
Regression between hunger and TD rates for LEF and HEF, separately. Scatter plot of the correlation between the hunger score and the degree of discounting (Log-*k* scores) for LEF (left panel) and HEF (right panel).

To clarify the effect of hunger on TD, we performed a regression analysis to determine the relation between the *baseline* hunger score as continuous dependent variable and the discount rates (*k*) for LEF and HEF as independent variables. We found that hunger was significantly associated with the HEF but not with the LEF (HEF: ß = 0.68 vs. LEF: ß = −0.27; p = 0.002) (Figure 4).

To better qualify this effect, we thereby applied a median split (e.g., [77]) on participants’ self-reported hunger rates (median = 2.5) to create groups of 20 low- (Low-hunger) and 20 high-hunger (High-hunger) participants thus comparing their TD rates for LEF and HEF. Hyperbolic *k* values were entered in an ANOVA, with group (Low-hunger, High-hunger) as between subject factor and food type (LEF, HEF) as within subjects factor. The analysis yielded no significant effect of group (F(1, 38) = 1.13, p = 0.29), no significant effect of food type (F(1, 38) = 2.44, p = 0.12), but a significant group×food type interaction (F(1, 38) = 7.91, p = 0.007). Post-hoc comparisons, performed with the Newman-Keuls test, indicated that, while Low-hunger participants discounted significantly less the HEF than the LEF (−1.165 vs. −0.6083, p = 0.01), the High-hunger participants did not differ between the HEF and the LEF (−0.4581 vs. −0.6169, p = 0.65). Note also that the difference between groups was limited to the HEF (Low-hunger: −1.165; High-hunger: −0.4581), whereas this was not the case of the LEF (Low-hunger: −0.6083; High-hunger: −0.6169).

In line with our hypothesis, participants showed reduced sensitivity for immediate amounts of reward (i.e., reduced TD rate) when faced with the food previously associated with a high number of errors compared with the food previously associated with a low number of errors. This effect was modulated by hunger. Specifically, the lower the hunger level, the less participants made impulsive choices when faced with the food paired with high-error rate, whereas, the higher the hunger state, the more participants showed the same degree of discounting for the two foods, suggesting that the two items were comparable in terms of motivational salience.

## Discussion

The present study investigated whether pairing food with errors would reduce impulsivity during food intertemporal choice, by enhancing the implementation of adaptive control strategies to promote more appropriate course of action. First, through a Stop-signal task, participants associated a different number of errors (low and high) with two different foods equally rated in the willingness to eat at the beginning of the experiment. Afterwards, they made choices between smaller-immediate and larger-later amounts of the two foods in two TD tasks, separately. Consistent with our hypothesis, foresighted food decisions were enhanced for the food previously associated with a high number of errors as compared with the food previously paired with a low number of errors. Moreover, the effect of reduced sensitivity for immediate amounts of food paired with high-error rate compared with the other food depended upon the hunger level of participants. Indeed, while less hungry individuals showed the expected reduced impatience when faced with the high-error food compared with the other food, hungrier individuals’ willingness to wait for future amounts of reward was comparable across the two foods after error manipulation. These findings suggest that, during decision-making for edible rewards, when experiencing high hunger, the error manipulation is not powerful enough in reducing the degree of discounting of future reward previously paired with high-error rate. Indeed, hunger increases the reinforcing value placed on food (e.g., [7], [50]–[56], [59]–[60], [79]–[82]), thereby increasing sensitivity for immediate amounts of reward (i.e., impulsivity). Thus, it might be that participants with high hunger were not able to make long-sighted choice toward the high-error food simply because this food and the low-error food were equally relevant for reducing their current state of need.

Why such errors-food pairing reduced impulsive food choice during intertemporal decisions? One possibility if that the present findings directly reflect inhibition rather than errors during the Stop-signal task, since the low-error condition is associated with successful inhibition, whereas the high-error condition is associated with unsuccessful inhibition. However, if successful inhibition can carry over from the Stop-signal task to intertemporal preference, there would have been decreased impulsive choice for the low-error food as compared with the high-error food. Conversely, our data indicate reduced discounting rate for the high-error food selectively (see also [48]). Moreover, the selective effect on high-error food rules out also the possibility that ego-depletion (e.g., [83]), negative mood [84], or induced stress or frustration [85] throughout the Stop-signal task explained our data, since we would have expected no difference in discount rate between the two foods.

Rather, we submit that errors serve as motivationally salient, warning signals that recruit cognitive control to match its anticipated demand [86]. During intertemporal choice, more visceral and automatic processes interact with more reasoned and rational mechanisms of future planning (e.g., [87]). The desire for sooner gratification competes with long-term gains maximization [9]–[11], [87]–[88], and increased sensitivity to immediate temptation is a core feature of impulsivity (e.g., [18]), landmark of several pathological conditions, including drug addiction and obesity (e.g., [18]–[27]). In order to obtain the larger outcome, it is necessary to carefully take into account the tradeoff between costs and benefits associated with options. In this context, errors might act in two ways (e.g., [89]). One possibility is that errors, as lapses in performance, represent warning signals that adaptively increase cognitive control in order to avoid further lapses in utility [36], [48]. Thus, in the present study, the high-error food cue, by enhancing the attention toward the increased likelihood of committing an error, would boost self-control over the decision [48], [89]–[90], thereby generating more long-sighted intertemporal choice for that food selectively.

A close related possibility is that, due to their aversive nature, errors [35] would act as teaching signals of avoidance learning against events associated with negative outcomes (i.e., loss in reward) [45]–[47], [91], which occurs through the generation of the ERN (e.g., [35]–[42], [91]–[92]) in the ACC (e.g., [43]). Thus, errors would activate adaptive adjustments in control processes that minimize costs in performance toward more efficient and controlled strategies and away from error-prone behavior [37]–[39], [91]. On this latter view, errors would act as somatic-markers guiding choice, namely, as anticipatory signals of negative outcome [93]–[96]. In the present study, errors committed during the Stop-signal task might have marked the high-error food so that this, in future encounters (i.e., during subsequent intertemporal choice), would have triggered adaptive self-control thus suppressing prepotent response (the choice of the tempting immediate reward) in favour of more advantageous course of actions (e.g., [97]). As such, in the present study, errors acted similarly as affective priming, which has the power of shaping even higher cognition as moral judgement (e.g., [98]–[99]). By inducing long-lasting carryover effect on the subsequent intertemporal choice task, errors induced more self-controlled decisions by warning against error-prone responses, thereby optimizing behavior. We thereby propose that the negative valence of errors alerts organisms toward performance monitoring and, thus, to when self-control is needed, as an *affect alarm model of control* [35], [39].

More broadly, it has been suggested that a prerequisite to exercise self-control when facing a temptation is to identify the conflict correctly [100]. For example, a dieter facing a delicious dessert in an isolated occasion is less likely to identify the conflict between her ultimate goal (i.e., losing weight) and the tempting high-caloric food, than when considering dessert for multiple similar future consumptions [100]. Thus, our modified version of the Stop-signal task might have induced subjects to face, in a brief lapse of time, multiple error signals and conflict concerning a specific food, thereby priming avoidance motivation for events associated with negative outcomes and more self-controlled decisions [39]–[40], [46]–[47].

Finally, since disordered eating behaviors have been recently linked consistently to drug addiction (e.g., [101]), sharing not only behavioral features like impulsivity and executive dysfunction, but also a documented dysregulation of the reward circuit (e.g., [3], [102]–[104]), we suggest to use the method tested here as a clinical training for people suffering from obesity and binge eating disorders. Similarly to a previous study in which alcohol-related stimuli were paired to stop trials in a Go-no-go task [105], thus increasing negative attitudes toward alcohol and reducing alcohol weekly consumption, here the effect of our error manipulation lasted for enough time to influence following intertemporal food decisions through indirect error-food pairing.

### Limitations

First, since recent findings highlighted that women and men have different behavioral (e.g., [57]–[58]) and neural (e.g., [59]–[61]) responses to food cues, hunger, and satiation, we tested female participants only, in order to control for sources of variability. Thus, we cannot generalize our findings to the population of healthy male young adults, and further research is needed to explore this issue. Moreover, although humans have been found to respond, both behaviorally and neurally, in a similar way to hypothetical and real rewards (e.g., [49], [67]–[68]), further investigation on the effect of error-food pairing should involve choices among real edible outcomes. This will help in avoiding the underestimation of several factors like visceral feelings of nausea and disgust (e.g., [106]), which may influence food decisions.

Second, we chose a Stop-signal task since it is a suitable paradigm to control for participants’ error rate. Indeed, we were allowed to induce all subjects making approximately the same number of errors in the low-error condition and in the high-error condition, respectively. Moreover, the low-error food served as control condition, since the adjustment we made after subject’s mistakes during the low-error condition (i.e., 5 ms) was negligible, and, as said before, this task is effective in augment cognitive control in the high-error condition, selectively [48]. However, the Stop-signal task used in the present study does not help to unravelling completely between error and inhibition effects on discounting rate, thus further studies involving error task which does not imply the implementation of inhibition is needed to state that one interpretation is more likely than the other (e.g., based on [107]–[109]).
